# Your suffering is also mine: Older and younger couples’ responses to the partner’s upsetting memory

**DOI:** 10.1093/geroni/igab046.3753

**Published:** 2021-12-17

**Authors:** Jared Isaac Cortez, Stephanie J Wilson, M Rosie Shrout, Janice Kiecolt-Glaser

**Affiliations:** 1 Southern Methodist University, Dallas, Texas, United States; 2 South Methodist University, Dallas, Texas, United States; 3 Purdue University, Purdue University, Indiana, United States; 4 Ohio State University, Columbus, Ohio, United States

## Abstract

Aging theories posit that older adults maximize their well-being by regulating their emotions and investing in their closest relationships. Most research has examined these mechanisms using study confederates rather than close dyads. The existing work on couples has focused on marital conflict; none has examined responses to the spouse’s emotional suffering. To address this, 107 married couples ages 40-86 listened to their partner disclose an upsetting personal memory. Afterward, listeners rated their own and their partner’s emotions and perspective-taking; observers reliably coded listeners’ engagement and disclosers’ emotional intensity. Aging theories offer competing predictions: older listeners may disengage from their partner’s disclosure to avoid experiencing negative emotions. Alternatively, older adults may be more engaged and thus more reactive, given the increased investment in their close relationships. Findings showed that older listeners rated their disclosing partner as less sad compared to younger counterparts (p < .05). However, this effect was attenuated (p = .077) by observed emotional intensity, as older disclosers exhibited less intense emotions. There were no age differences in listeners’ own reactivity, perspective-taking, or observed engagement. Taken together, older adults disclosed with less emotional intensity, consistent with theory. By contrast, older listeners’ ratings were validated by external coders, not driven by positivity biases. Further, older listeners were no more or less engaged or reactive to their spouse’s disclosure than younger listeners. This study highlights a context wherein social and emotional motivations are at odds. Teasing these motivations apart will help us to better understand how social-emotional processes develop across adulthood.

